# Evaluation of overall survival and barriers to surgery for patients with breast cancer treated without surgery: a National Cancer Database analysis

**DOI:** 10.1038/s41523-021-00294-w

**Published:** 2021-07-05

**Authors:** D. Boyce-Fappiano, I. Bedrosian, Y. Shen, H. Lin, O. Gjyshi, A. Yoder, S. F. Shaitelman, W. A. Woodward

**Affiliations:** 1 Departments of Radiation Oncology, The University of Texas MD Anderson Cancer Center, Houston, TX USA; 2 Departments of Breast Surgical Oncology, The University of Texas MD Anderson Cancer Center, Houston, TX USA; 3 Departments of Biostatistics, The University of Texas MD Anderson Cancer Center, Houston, TX USA

**Keywords:** Breast cancer, Outcomes research

## Abstract

Surgery remains the foundation of curative therapy for non-metastatic breast cancer, but many patients do not undergo surgery. Evidence is limited regarding this population. We sought to assess factors associated with lack of surgery and overall survival (OS) in patients not receiving breast cancer surgery. Retrospective cohort study of patients in the US National Cancer Database treated in 2004–2016. The dataset comprised 2,696,734 patients; excluding patients with unknown surgical status or stage IV, cT0, cTx, or pIS, metastatic or recurrent disease resulted in 1,192,294 patients for analysis. Chi-square and Wilcoxon rank-sum tests were used to assess differences between groups. OS was analyzed using the Kaplan–Meier method with a Cox proportional hazards model performed to assess associated factors. In total 50,626 (4.3%) did not undergo surgery. Black race, age >50 years, lower income, uninsured or public insurance, and lower education were more prevalent in the non-surgical cohort; this group was also more likely to have more comorbidities, higher disease stage, and more aggressive disease biology. Only 3,689 non-surgical patients (7.3%) received radiation therapy (RT). Median OS time for the non-surgical patients was 58 months (3-year and 5-year OS rates 63% and 49%). Median OS times were longer for patients who received chemotherapy (80 vs 50 (no-chemo) months) and RT (85 vs 56 (no-RT) months). On multivariate analysis, age, race, income, insurance status, comorbidity score, disease stage, tumor subtype, treatment facility type and location, and receipt of RT were associated with OS. On subgroup analysis, receipt of chemotherapy improved OS for patients with triple negative (HR 0.66, 95% CI 0.59–0.75, *P* < 0.001) and HER2^+^ (HR 0.74, 95% CI 0.65–0.84, *P* < 0.001) subgroups while RT improved OS for ER^+^ (HR 0.72, 95% CI 0.64–0.82, *P* < 0.001) and favorable-disease (ER+, early-stage, age >60) (HR 0.61, 95% CI 0.45–0.83, *P* = 0.002) subgroups. Approximately 4% of women with breast cancer do not undergo surgery, particularly those with more aggressive disease and lower socioeconomic status. Despite its benefits, RT was underutilized. This study provides a benchmark of survival outcomes for patients who do not undergo surgery and highlights a potential role for use of RT.

## Introduction

Current therapy for breast cancer (BC) most often involves a multimodality approach, with neoadjuvant systemic therapy, surgical resection, and adjuvant radiation therapy (RT) or chemotherapy/hormonal therapy considered on the basis of tumor- and patient-related risk factors^1^. Surgery remains the foundation of curative therapy for patients with local-regional disease; 5-year overall survival (OS) rates after surgery for localized disease are 99% and, for regional disease, 85%^2^.

No recommendations or standard of care have been established for patients with BC who do not undergo surgery^1^. Several studies have evaluated the role of definitive RT with or without systemic therapy for patients who do not undergo surgery; local-regional control rates after this therapy have ranged from 50% to 96%^3–11^. This approach has been used mostly for elderly women, the group that’s most likely to be offered a non-surgical approach for BC^12,13^. The use of stereotactic body radiation therapy (SBRT), which allows delivery of highly conformal and ablative radiation doses, has also been explored as definitive treatment or as an additional boost to conventional RT, with promising early results^6^. Nevertheless, surgery still remains the pillar of successful BC treatment. To date, factors linked with lack of surgery include advanced disease, older age, comorbidities, access to care, and patient preference^12–17^.

The limited evidence regarding factors associated with not receiving surgery and oncologic outcomes after such therapy, and the lack of prospective trials, consensus guidelines, or recommended treatment strategies for such patients led us to investigate these gaps on a population level by analyzing the US National Cancer Data Base (NCDB).

## Results

### Patient characteristics and patterns of care

**Fig. 1:**
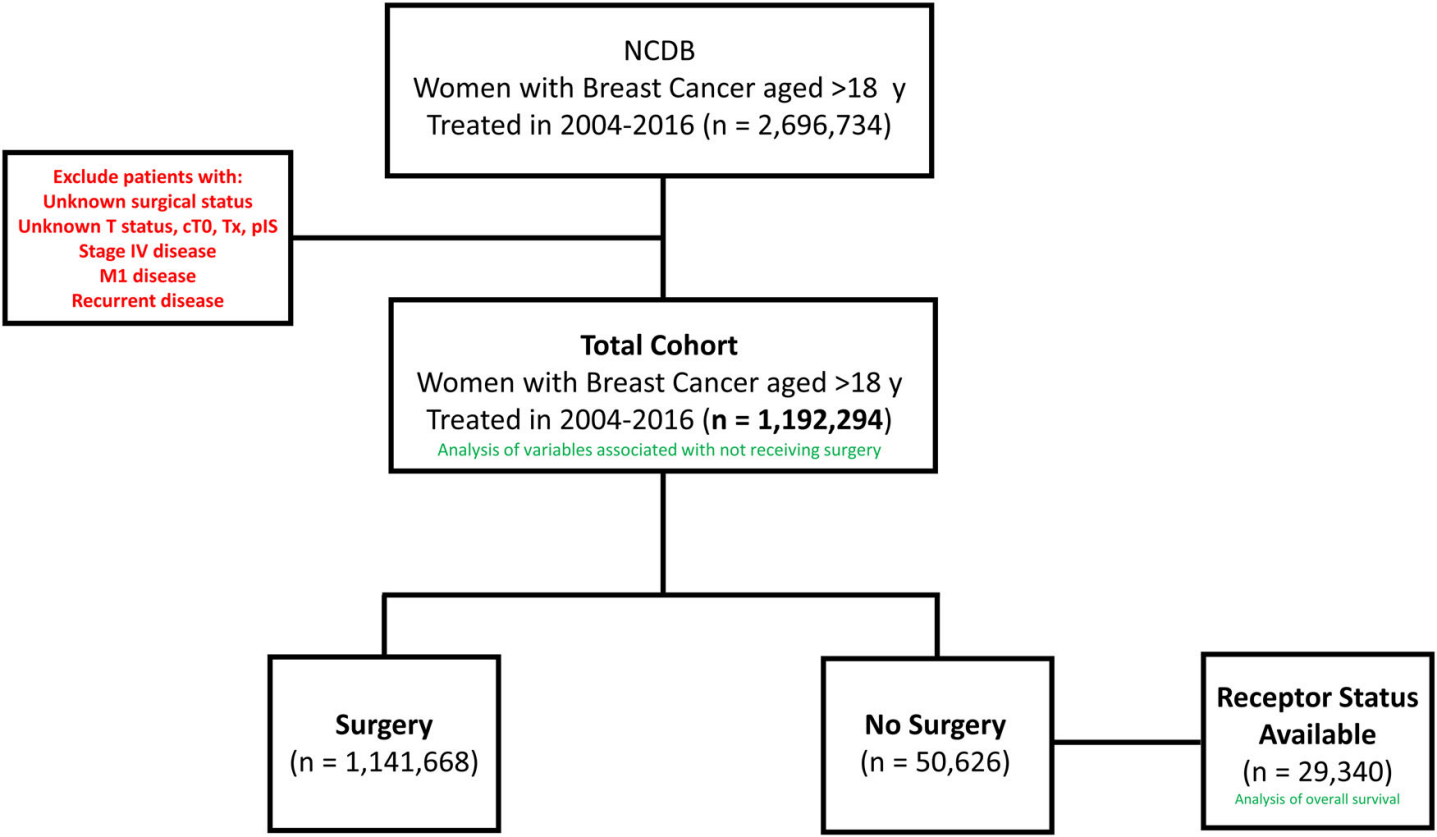
CONSORT diagram. Total screened population, exclusion criteria (red), total analyzed cohort, and the overall survival analysis subcohort with available receptor status are displayed.

To examine variables associated with not undergoing surgery, we analyzed 1,192,294 patients with BC, of whom 50,626 (4.3%) did not undergo surgery (Fig. 1). Evaluating the trend of no-surgery overtime there is a significant correlation as patients who did not undergo surgery decreased from 4.7% in 2004 to 3.5% in 2009, and then increased from 3.5% in 2009 to 5.3% in 2016 (*P* < 0.001). Reasons for not receiving surgery included not being planned as part of initial treatment (55.6%), refusal by the patient and/or family member (13.6%), recommended but unknown if performed (10.9%), unknown if surgery was recommended or performed (7.7%), contraindicated due to patient risk factors (7.7%), recommended but not performed without an identifiable reason (2.9%), and surgery was recommended but patient was deceased prior to having surgery (1.5%).

Differences noted between patients who did and did not receive surgery are listed in Table 1. In terms of demographics, lack of surgery was associated with patients being Black (18.7% vs 11.1%, *P* < 0.001), over 50 years old (78.4% vs 74.3%, *P* < 0.001), having lower income (i.e., earning < $30,000 per year; 14.1% vs 10.5%, *P* < 0.001), having public (Medicare or Medicaid) insurance (54.8% vs 42.2%, *P* < 0.001) or being uninsured (4.3% vs 1.9%, *P* < 0.001), living in a zip code in which >29% of residents do not have a high school degree [19% vs 13.6%, *P* < 0.001), living within a metro area (86.2% vs 82.7%, *P* < 0.001), living in the southern US (40.4% vs 34.1%, *P* < 0.001) and receiving treatment at an academic facility (35% vs 28.2%, *P* < 0.001). Further exploration revealed a trend towards declining use of surgery by patient age decade with non-surgical rates of 3.6% < 50 y, 3.2% ≥50 - <60 y, 3.1% ≥60 - <70 y, 3.9% ≥70 - <80 y, and 12.8% ≥80 y (*P* < 0.001).With regard to clinical factors, lack of surgery was more likely for patients with more comorbidities (i.e., Charlson/Deyo comorbidity score of 2–3 [5.4% vs 3.2%, *P* < 0.001]), those with clinical stage II disease (42% vs 32.4%, *P* < 0.001) or stage III disease (25.1% vs 7.9%, *P* < 0.001), triple-negative (9.2% vs 8.4%, *P* < 0.001) or HER2^+^ (11.3% vs 9.7%, *P* < 0.001) subtypes, and those who did not receive RT (90.3% vs 35.2%, *P* < 0.001), chemotherapy (64.5% vs 52.2%, *P* < 0.001), or hormone therapy (62.6% vs 29.3%, *P* < 0.001). Looking specifically at patients with stage I disease (*n* = 698,442, 16,654 without surgery) we found similar trends to the overall cohort with age, Black race, insurance status (public or uninsured), Charlson/Deyo comorbidity score (3), residing in an urban area, living in the Midwest, having higher grade disease, and lack of receipt of other oncologic therapy (chemotherapy, hormone therapy & RT) significantly associated with a higher probability of not having surgery”.

**Table 1:** Patient, tumor, and treatment characteristics.

Variable	Surgery (*n* = 1,141,668)	No surgery (*n* = 50,626)	*P* Value
*Age, Years*			<0.001
≤50	293,330 (25.7)	10,940 (21.6)	
>50	848,338 (74.3)	39,686 (78.4)	
*Race/ethnicity*			<0.001
White	954,520 (83.6)	37,651 (74.4)	
Black	125,306 (11.0)	9,297 (18.4)	
American Indian, Aleutian, or Eskimo	3,297 (0.3)	143 (0.3)	
*Eskimo*			
Chinese	5,930 (0.5)	272 (0.5)	
Japanese	3,191 (0.3)	117 (0.2)	
Filipino	6,335 (0.6)	241 (0.5)	
Hawaiian	1,276 (0.1)	40 (0.1)	
Korean	2,391 (0.2)	130 (0.3)	
Vietnamese	2,150 (0.2)	89 (0.2)	
Laotian	193 (0.0)	4 (0.0)	
Hmong	49 (0.0)	6 (0.0)	
Kampuchean	197 (0.0)	16 (0.0)	
Thai	323 (0.0)	16 (0.0)	
Asian Indian or Pakistani, NOS	3,811 (0.3)	189 (0.4)	
Asian Indian	2,194 (0.2)	106 (0.2)	
Pakistani	293 (0.0)	23 (0.0)	
Micronesian, NOS	52 (0.0)	7 (0.0)	
Chamorran	10 (0.0)	0 (0.0)	
Guamanian, NOS	47 (0.0)	3 (0.0)	
Polynesian, NOS	19 (0.0)	1 (0.0)	
Tahitian	4 (0.00)	0 (0.0)	
Samoan	160 (0.0)	13 (0.0)	
Tongan	47 (0.0)	2 (0.0)	
Melanesian, NOS	6 (0.0)	0 (0.0)	
Fiji Islander	42 (0.0)	1 (0.0)	
New Guinean	7 (0)	2 (0)	
Other Asian, Asian and Oriental, NOS	9,249 (0.8)	498 (1)	
Pacific Islander, NOS	656 (0.1)	37 (0.1)	
Other	9,302 (0.8)	733 (1.4)	
Unknown	10,611 (0.9)	989 (2)	
*CDC Score*			<0.001
0	960,904 (84.2)	43,120 (85.2)	
1	143,985 (12.6)	4,792 (9.5)	
2	27,608 (2.4)	1,719 (3.4)	
3	9,171 (0.8)	995 (2)	
*Median Income, USD*			<0.001
< $30,000	119,919 (10.5)	7,145 (14.1)	
$30,000-34,999	173,260 (15.2)	7,871 (15.5)	
$35,000-45,999	295,042 (25.8)	12,620 (24.9)	
≥$46,000	516,613 (45.3)	21,264 (42)	
Unknown	36,834 (3.2)	1,726 (3.4)	
*No HSD Quartile*			<0.001
≥29%	155,278 (13.6)	9,625 (19)	
20–28.9%	233,546 (20.5)	11,020 (21.8)	
14–19.9%	256,442 (22.5)	10,280 (20.3)	
<14%	459,442 (40.2)	17,966 (35.5)	
Unknown	36,960 (3.2)	1,735 (3.4)	
*Insurance status*			<0.001
Public insurance	481,574 (42.2)	27,729 (54.8)	
Private insurance	622,152 (54.5)	17,130 (33.8)	
Uninsured	22,106 (1.9)	2,191 (4.3)	
Unknown	15,836 (1.4)	3,576 (7.1)	
*Residential area*			<0.001
Metro	944,529 (82.7)	43,640 (86.2)	
Urban	148,071 (13)	5,018 (9.9)	
Rural	19,166 (1.7)	634 (1.3)	
Unknown	29,902 (2.6)	1,334 (2.6)	
*Treatment facility*			<0.001
Academic	322,042 (28.2)	17,700 (35)	
Community CC	261,029 (22.9)	11,275 (22.3)	
Comprehensive CP	494,735 (43.3)	18.606 (36.8)	
Unknown	63,862 (5.6)	3,045 (6)	
*Facility location*			<0.001
Midwest	278,559 (24.4)	10,113 (20)	
Northeast	229,200 (20.1)	10,507 (20.8)	
South	389,706 (34.1)	20,439 (40.4)	
West	180,341(15.8)	6,522 (12.9)	
Unknown	63,862 (5.6)	3,045 (6)	
*TNM stage, clinical*			<0.001
1	681,788 (59.7)	16,654 (32.9)	
2	370,103 (32.4)	21,249 (42)	
3	89,777 (7.9)	12,723 (25.1)	
*Grade*			<0.001
1	248,905 (21.8)	7,635 (15.1)	
2	474,487 (41.6)	18,621 (36.8)	
3	354,452 (31)	15,930 (31.5)	
4	3,102 (0.3)	204 (0.4)	
9	60,722 (5.3)	8,236 (16.3)	
*Estrogen receptor*			<0.001
Negative	210,682 (18.5)	10,741 (21.2)	
Positive	913,107 (80)	35,513 (70.1)	
Unknown	17,879 (1.6)	4,372 (8.6)	
*Progesterone receptor*			<0.001
Negative	319,938 (28)	15,673 (31)	
Positive	802,273 (70.3)	30,029 (59.3)	
Unknown	19,457 (1.7)	4,924 (9.7)	
*Hormone receptor status*			<0.001
Negative	199,047 (17.4)	9,894 (19.5)	
Positive	924,653 (81)	36,185 (71.5)	
Unknown	17,968 (1.6)	4,547 (9%)	
*HER2*			<0.001
Negative	666,640 (58.4)	26,332 (52)	
Positive	110,512 (9.7)	5,702 (11.3)	
Unknown	364,516 (31.9)	18,592 (36.7)	
*Triple-negative*			<0.001
No	957,262 (83.8)	38,063 (75.2)	
Yes	95,740 (8.4)	4,663 (9.2)	
Unknown	88,666 (7.8)	7,900 (15.6)	
*Tumor subtype*			<0.001
Luminal A/B	570,580 (50)	21,612 (42.7)	
Triple Negative	95,740 (8.4)	4,663 (9.2)	
HER2+	110,512 (9.7)	5,702 (11.3)	
Unknown	364,836 (32)	18,649 (36.8)	
*Chemotherapy*			<0.001
Yes	520,741 (45.6)	14,581 (28.8)	
No	595,801 (52.2)	32,636 (64.5)	
Unknown	25,126 (2.2)	3,409 (6.7)	
*Radiation therapy*			<0.001
Yes	733,070 (64.2)	3,689 (7.3)	
No	402,016 (35.2)	45,704 (90.3)	
Unknown	6,582 (0.6%)	1,233 (2.4)	
*Hormone therapy*			<0.001
Yes	772,977 (67.7)	14,953 (29.5)	
No	334,160 (29.3)	31,705 (62.6)	
Unknown	34,531 (3)	3,968 (7.8)	

*NOS* Not other specific, *CDC* Charlson/Deyo Comorbidity, *USD* United States dollar, *HSD* high school degree, *CC* Cancer Center, *CP* Cancer Program, Grade 4 = Undifferentiated, anaplastic, Grade 9 = unknown.

Finally, of the entire cohort 19,107 (1.6%) patients received no therapy at all compared to 1,111,800 (93.2%) patients who received some form of treatment (surgery, chemotherapy, radiation therapy, or hormone therapy). Surprisingly, Charlson/Deyo comorbidity score was inversely associated with receipt of any treatment (*P* < 0.001) as patients without any comorbidities were more likely to not receive any therapy (44.4%) than those with one or more comorbidities (32.7–34.1%) (*P* < 0.001).

Patterns of care for the 50,626 patients who did not undergo surgery for BC in 2004–2016 are shown in Supplementary Table 1. Trimodality therapy (chemotherapy, RT, and hormone therapy) was provided to 1.4% of patients; 28.8% of patients received chemotherapy, 29.5% received hormone therapy, and only 7.3% received RT. On multivariate analysis, older age (odds ratio [OR] 0.986, 95% confidence interval [CI] 0.983–0.989, *P* < 0.0001), having more comorbidities (OR 0.73, 95% CI 0.61–0.88, *P* = 0.0011), being uninsured (OR 0.73, 95% CI 0.60–0.89, *P* = 0.0015) compared to having private insurance, having being treated in the western (OR 0.77, 95% CI 0.69–0.87, *P* < 0.0001) or northeastern US (vs south) (OR 0.86, 95% CI 0.78–0.95, *P* = 0.0032) were associated with decreased use of RT among patients not undergoing surgery (Supplementary Table 2).

### Overall survival analysis

To examine OS, patients who received non-surgical treatment for BC in 2010–2015 and whose BC receptor subtype status was known were examined to account for receptor subtype surrogates (*n* = 29,340; Fig. 1). Median follow-up time for this subcohort was 34 months (95% CI 33–35). The median OS time for these patients was 58 months (95% CI 56–60), and 3-year and 5-year OS rates were 63% and 49%. Significantly improved OS was noted among patients who received chemotherapy (median OS time 80 vs 50 (not receiving chemo) months; 3-year OS 66%; 5-year OS 56% vs 45%, *P* < .0001,) or RT (median OS time 85 vs 56 (not receiving RT) months; 3-year OS 70%; 5-year OS 59% vs 48%, *P* < .0001), but OS was significantly poorer for patients receiving endocrine therapy (median OS time 41 vs 80 (not receiving HT) months; 3-year OS 56%; 5-year OS 36% vs 56%, *P* < .0001). Several factors were found to be statistically significant for OS on multivariate analysis (Table 2); specifically, sociodemographic factors such as age (for per-year increase, hazard ratio [HR] 1.034, 95% CI 1.032–1.036, *P* < 0.001); race (for non-Black other vs White, HR 0.75, 95% CI 0.65–0.86 *P* < 0.001); annual income (for < $30,000 vs ≥ $46,000, HR 1.14, 95% CI 1.06–1.22, *P* < 0.001), and uninsured (HR 1.32, 95% CI 1.14–1.52, *P* < 0.001) or public insurance (HR 1.34, 95% CI 1.25–1.44, *P* < 0.001) vs private insurance were significant. Regarding medical factors, Charlson/Deyo comorbidity score (for 3 vs 0, HR 2.36, 95% CI 2.10–2.67, *P* < 0.001; for 2 vs 0, HR 1.88, 95% CI 1.72–2.05, *P* < 0.001; for 1 vs 0, HR 1.42, 95% CI 1.34–1.51, *P* < 0.001), TNM disease stage (for III vs I, HR 2.60, 95% CI 2.44–2.78, *P* < 0.001; for II vs I, HR 1.66, 95% CI 1.56–1.76, *P* < 0.001), and tumor subtype (for HER2^+^ vs Luminal A/B, HR 1.19, 95% CI 1.12–1.28, *P* < 0.001; for triple-negative vs Luminal A/B, HR 1.76, 95% CI 1.64–1.89, *P* < 0.001) were significant. Finally, treatment characteristics found to be significant included facility type (for comprehensive cancer program vs academic facility, HR 1.25, 95% CI 1.18–1.32, *P* < 0.001; for community center vs academic facility, HR 1.17, 95% CI 1.10–1.25, *P* < 0.001), facility location (midwestern vs southern US, HR 1.26, 95% CI 1.19–1.34, *P* < 0.001), and receipt of RT (yes vs no, HR 0.73, 95% CI 0.67–0.81, *P* < 0.001).

**Table 2:** Multivariate analysis of factors associated with overall survival.

Variable reference	Comparison	Hazard ratio	95% confidence interval	*P* value
Age, years				<0.001
Per-year increase	Continuous	1.034	1.032–1.036	
Race				0.0003
White	Black	1.01	0.95–1.08	0.70
	Other	0.75	0.65–0.86	<0.0001
CDC Score				<0.001
0	3	2.36	2.10–2.67	<0.001
	2	1.88	1.72–2.05	<0.001
	1	1.42	1.34–1.51	<0.001
Median Income, USD				<0.001
≥$46,000	<$30,000	1.14	1.06–1.22	0.0006
	$30,000–34,999	1.13	1.06–1.21	0.0003
	$35,000–45,999	1.13	1.07–1.20	<0.0001
Treatment facility				<0.001
Academic	Comprehensive CP	1.25	1.18–1.32	<0.0001
	Community CC	1.17	1.10–1.25	<0.0001
Facility location				<0.001
South	West	1.02	0.94–1.09	0.70
	Northeast	0.99	0.93–1.05	0.73
	Midwest	1.26	1.19–1.34	<0.0001
Insurance status				<0.001
Private Insurance	Uninsured	1.32	1.14–1.52	0.0002
	Public Insurance	1.34	1.25–1.44	<0.0001
TNM group stage				<0.001
1	3	2.60	2.44–2.78	<0.001
	2	1.66	1.56–1.76	<0.001
Tumor phenotype				<0.001
Luminal A/B	HER2+	1.19	1.12–1.28	<0.001
	Triple negative	1.76	1.64–1.89	<0.001
Radiation therapy				<0.001
No	Yes	0.73	0.67–0.81	

*P* value is for overall effect of each variable.

*CDC* Charlson/Deyo Comorbidity, *USD* United States dollar, *HSD* high school degree, *CC* Cancer Center, *CP* Cancer Program.

**Fig. 2:**
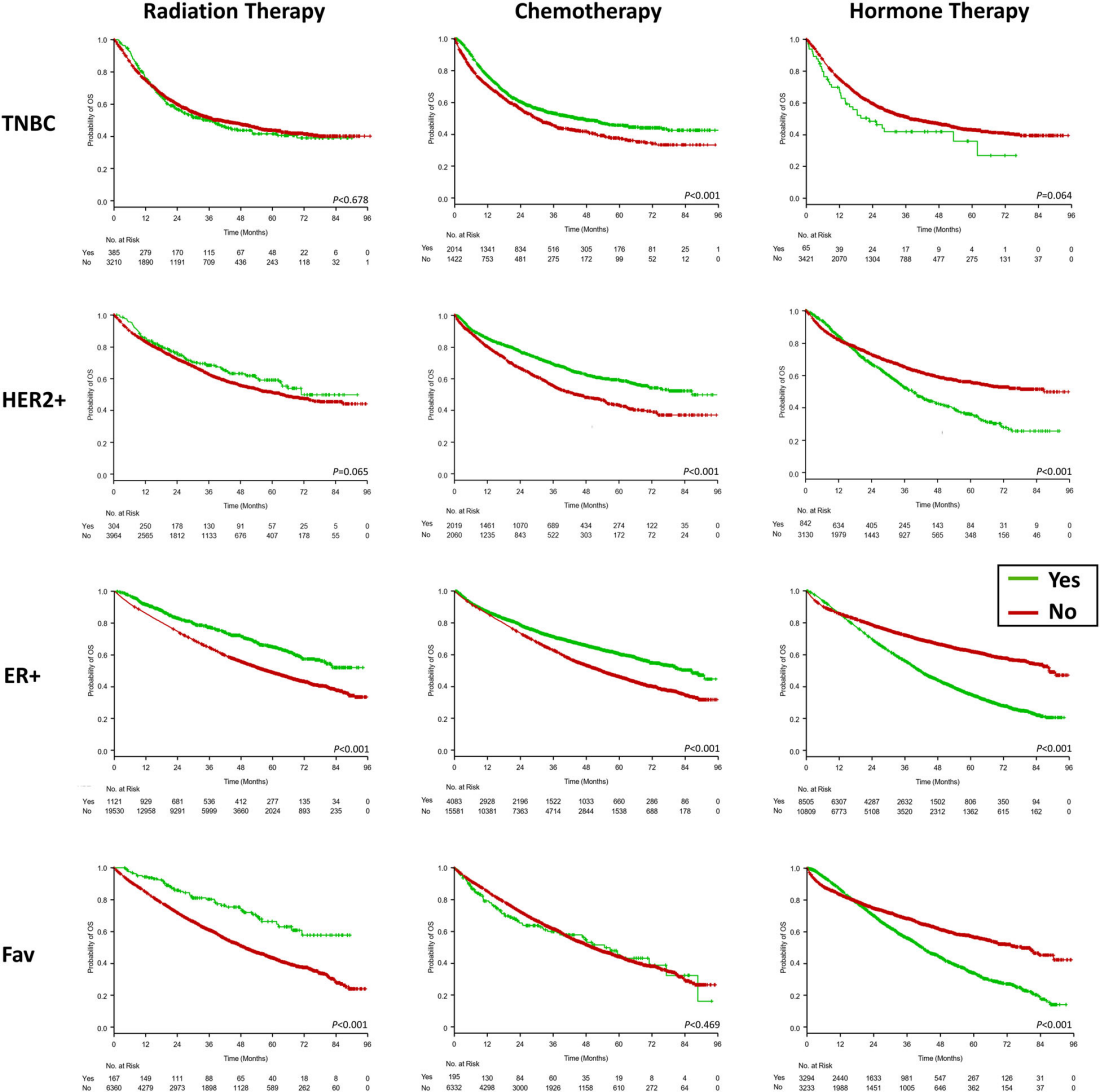
Kaplan–Meier Curves for overall survival. Kaplan-Meier curves for overall survival are shown by tumor subtype (TNCB, HER2 +, Luminal A/B, and Favorable) and by treatment type (radiation therapy, chemotherapy, and hormone therapy).

A subgroup analysis was conducted for patients with triple-negative breast cancer (TNBC), HER2^+^ cancer, ER^+^, or ‘favorable’ disease (i.e., that likely to be curable with RT [ER^+^, HER2^–^, cT1-2, cN0, age >60 years]) to assess whether RT chemotherapy, or hormone therapy was associated with differences in OS in these subgroups (Fig. 2). On multivariate analysis, older age, higher Charlson/Deyo comorbidity score, and advanced TNM disease stage were associated with worse OS in all subgroups (Table 3). Receipt of chemotherapy was associated with improved OS in the TNBC (HR 0.66, 95% CI 0.59–0.75, *P* < 0.001) and HER2^+^ (HR 0.74, 95% CI 0.65–0.84, *P* < 0.001) subgroups, whereas receipt of RT was associated with improved OS in the ER^+^ (HR 0.72, 95% CI 0.64–0.82, *P* < 0.001) and favorable-disease (HR 0.61, 95% CI 0.45–0.83, *P* = 0.002) subgroups. Interestingly, the use of hormone therapy was not independently associated with survival in any subgroup, including those with ER^+^ disease.

**Table 3:** Multivariate analysis of factors associated with overall survival analysis by breast cancer subgroup.

Variable	Comparison	TNBC	HER2 +	Luminal A/B	Favorable
reference		HR (95% CI) *P* value	HR (95% CI) *P* value	HR (95% CI) *P* value	HR (95% CI) *P* value
Age, years					
Per-year increase	continuous	1.02 (1.02–1.03) <0.0001	1.03 (1.03–1.04) <0.0001	1.03 (1.03–1.04) <0.0001	1.05 (1.04–1.05) <0.0001
Race					
White	Black	0.88 (0.78–0.99) 0.038	N/A	1.02 (0.95–1.1) 0.540	0.97 (0.85–1.11) 0.6800
	Other	0.77 (0.56–1.04) 0.086	N/A	0.67 (0,57–0.79) <0.0001	0.62 (0.46–0.85) 0.0024
CDC Score					
0	3	2.94 (2.10–4.12) <0.0001	2.35 (1.70–3.23) <0.0001	2.38 (2.10–2.71) <0.0001	2.84 (2.34–3.44) <0.0001
	2	2.19 (1.75–2.74) <0.0001	1.67 (1.31–2.13) <0.0001	1.88 (1.71–2.06) <0.0001	2.16 (1.86–2.50) <0.0001
	1	1.41 (1.21–1.65) <0.0001	1.36 (1.16–1.58) 0.0001	1.44 (1.34–1.54) <0.0001	1.62 (1.45–1.81) <0.0001
Median Income, USD					
≥$46,000	<$30,000	1.14 (0.97–1.34) 0.1240	N/A	1.29 (1.16–1.43) <0.0001	1.27 (1.06–1.51) 0.0084
	$30,000–34,999	1.25 (1.08–1.45) 0.0037	N/A	1.19 (1.09–1.30) <0.0001	1.26 (1.09–1.45) 0.0016
	$35,000–45,999	1.20 (1.04–1.37) 0.0110	N/A	1.18 (1.10–1.26) <0.0001	1.16 (1.04–1.30) 0.0100
No HSD quartile					
≥1:29%	<14%	N/A	N/A	1.24 (1.12–1.38) <0.0001	1.32 (1.11, 1.57) 0.0019
	14%-19.9%	N/A	N/A	1.23 (1.11–1.35) <0.0001	1.34 (1.13, 1.58) 0.0008
	20%-28.9%	N/A	N/A	1.19 (1.09–1.30) 0.00020	1.26 (1.08, 1.47) 0.0031
Insurance status					
Private insurance	Uninsured	1.21 (0.94–1.54) 0.1330	1.56 (1.20–2.05) 0.00110	1.23 (1.02–1.47) 0.0280	N/A
	Public Insurance	1.30 (1.13–1.49) 0.0002	1.54 (1.32–1.80) <0.0001	1.40 (1.29–1.52) <0.0001	N/A
Clinical T status					
T1	T2	N/A	N/A	N/A	1.44 (1.33–1.57) <0.0001
TNM disease stage					
1	3	5.15 (4.24–6.26) <0.0001	2.68 (2.24–3.22) <0.0001	2.35 (2.19–2.50) <0.0001	N/A
	2	2.03 (1.67–2.46) <0.0001	1.62 (1.36–1.94) <0.0001	1.67 (1.56–1.78) <0.0001	N/A
Hormone receptor status					
Negative	Positive	N/A	0.86 (0.76–0.99) 0.029	N/A	N/A
Chemotherapy					
No	Yes	0.66 (0.59–0.75) <0.0001	0.74 (0.65–0.84) <0.0001	N/A	N/A
Radiation therapy					
No	Yes	N/A	N/A	0.72 (0.64–0.82) <0.0001	0.61 (0.45–0.83) 0.0016

*TNBC* triple-negative breast cancer, Favorable, ER + cT1-T2 cN0 age > 60 years; *CDC* Charlson/Deyo Comorbidity, *USD* United States dollar, *HSD* high school degree, *CC* Cancer Center, *CP* Cancer Program, *N/A* not available (i.e., not included in multivariate analysis).

Finally, we performed an exploratory analysis comparing survival outcomes for non-surgical patients who received hormone therapy compared to those who received any other treatment (chemotherapy and or radiation therapy) or no treatment at all. Surprisingly the cohort receiving endocrine therapy alone had inferior OS than those who received any other treatments (chemotherapy and/or radiation therapy) or those who didn’t receive any treatment (*p*-Value < 0.001) with median OS of 38.1, 75.9, and 75.0 months respectively.

## Discussion

This study represents the largest analysis of women who did not undergo surgical resection as part of therapy for breast cancer, and is the first to look at outcomes for these patients according to subtype. Overall, 4.3% women in the United States did not receive surgery for non-metastatic breast cancer. When comparing patients who did vs did not have surgery, our findings suggest that several oncologic and socioeconomic factors influence the likelihood of having surgery; having lower socioeconomic status and more advanced or complicated disease translated into being less likely to receive surgery. Further, the survival outcomes among patients treated without surgery highlight the potential value of RT when surgery cannot be used. Indeed, RT among such patients seems to be grossly underused, with only 7.3% of patients receiving it, despite being associated with improved OS.

Socioeconomic factors have been previously reported to be major barriers to accessing care for BC. The influence of financial toxicity on patients’ decisions regarding breast surgery has been evaluated. Women who make ≤$45,000/year have been shown to prioritize the cost of treatment over breast preservation or appearance^18^. Moreover, about one-quarter to one-third of women with BC experience significant financial hardship from treatment costs^18,19^. While evidence suggests that surgical costs are on the order of approximately 5–10% less than that of RT for breast cancer, adjuvant RT has been shown to be highly cost-effective thus suggesting a possible value-driven approach for patients who cannot receive or decline surgery^20,21^. Insurance status has been shown to be associated with advanced disease stage at diagnosis and poorer OS among patients with BC^22,23^, with one report of increased risks of mortality of 49% for uninsured patients and 40% for patients with Medicaid^23^. Notably, insurance status and out-of-pocket costs have also been shown to correlate with not receiving surgery for lung cancer^24^. Cost clearly affects a patient’s decision to proceed with treatment, and based on this evidence the costs associated with surgery may be a deterrent for low-middle class women. Minority women may be particularly vulnerable because of superimposed access-to-care issues. This sentiment is reiterated by Jagsi and colleagues in a report showing minority women with BC to be at disproportionately high risk of financial toxicity^19^. Further, evidence has shown that Black women are more prone to experiencing treatment delays, not following treatment recommendations, or declining treatment, all of which significantly affect mortality^22,23^. Education level can also affect a patient’s ability to comply with treatment and participate in early screening. Women with less education have been shown to present with more advanced disease, which in turn may affect candidacy for surgery. A large population study from China reported that women who attended university (vs women with no formal education) were much less likely to present with locally advanced disease (17.7% vs 31.5%)^25^. This finding probably stems from participation in screening programs, as a large meta-analysis revealed that women of the highest education level were much more likely to undergo annual mammography, and women of low educational status were significantly less likely to follow screening recommendations^26^. Additionally, it is important to highlight the role of the healthcare provider and the presence of implicit bias and systemic racism that may negatively impact access and or treatment recommendations for minority patients^27^. Overall there is an abundance of evidence in both oncologic and surgical series that show significant healthcare disparities affecting black and minority patients while controlling for other socioeconomic factors^28–33^. In summary, these findings highlight the importance of identifying patients with limited financial resources, those with lower education status, and those who are of racial minorities early in the care cycle to provide additional social support to minimize potential financial toxicity and to provide early education to help facilitate informed decision-making about BC detection and treatment. This also highlights the importance of acknowledging the reality of implicit bias in healthcare providers and working towards educated a more diverse and culturally competent generation of physicians.

Beyond socioeconomic status, several clinical factors also were found to be associated with not receiving surgery. Both advanced age and the presence of comorbidities can influence adherence to standard treatment protocols among patients with non-metastatic BC, including the choice of definitive and adjuvant therapy^12,34^. In contrast, the presence of higher disease stage and more aggressive disease biology in patients who do not have surgery probably corresponds with subsequent delays in care related to the aforementioned socioeconomic barriers that may precipitate progression of disease, thereby precluding surgery^35^. This concept is unfortunately enhanced for Black women, who are 4–5 times more likely to incur delays in excess of 60 days in BC management, resulting in significantly less surgical-directed therapy (7.5% vs 1.5%) relative to White women^36^. Discontinuation of therapy can also influence surgical status, particularly for minority women receiving neoadjuvant therapy before surgery. A secondary analysis of the SWOG S8814/S8897 trials found that Black women, despite having more aggressive disease features, were more likely to have early discontinuation of cancer-directed therapy (11% vs 7%), resulting in inferior oncologic outcomes^37^. Again, these findings emphasize the importance of providers being aware and identifying patients with such vulnerabilities to minimize possible delays in care and maximize adherence to optimal cancer-directed therapy.

While the survival outcomes reported in this series are inferior to historical survival rates for patients with BC, it is important to acknowledge that these patients had more advanced disease, with more aggressive features, greater comorbidities, and lower socioeconomic status. Interestingly, prior studies evaluating the use of definitive RT alone or in combination with systemic therapy have shown highly variable outcomes, with 3- to 5-year OS rates ranging from 38% to 95%^3–6,8–11^. A Belgian study was one of the first to evaluate definitive RT for operable BC and reported 5-year and 10-year OS rates of 67% and 29%^10^. Interestingly, that study found that radiation dose was the strongest predictor of local disease control, with higher doses required to reach local control outcomes comparable to those after surgical resection^10^. The benefit of combined chemoradiation for the definitive management of BC was reported in a series of 250 patients managed with neoadjuvant chemotherapy with 5-year OS rates of 95% for those with stage I disease, 94% for stage IIA, 80% for stage IIB, 60% for stage IIIA, and 58% for stage IIIB disease^11^. These findings highlight the importance of using multimodality therapy when surgery is not used. Interestingly, our subgroup analysis showed that patients with more aggressive disease biology (TNBC and HER2^+^) derived a benefit from chemotherapy whereas those with ER^+^ or favorable disease derived an exclusive benefit from RT. These findings are intuitive given the higher rates of metastatic disease for HER2^+^ and TNBC subtypes, which make chemotherapy the ideal treatment approach^38^. The poorer outcomes observed on univariate analysis of hormonal therapy in our analysis seem to be related to patient heterogeneity, as the use of hormones was not an independent predictor of worse outcome on multivariable analysis. While patients receiving hormone therapy alone were noted to have inferior OS compared to those receiving any other treatment or no treatment at all we suspect this finding is most likely due to the influence of confounding factors and/or selection bias Similar findings were observed in a series evaluating definitive hormone therapy for women aged >75 years, which reported that 35% of patients developed progressive disease suggesting that hormone therapy may not be the optimal therapeutic option for patients who do not undergo surgery^39^. Another possible explanation of the inferior OS in such patients is that considering hormone therapy may offer little benefit in non-surgical patients with the gross disease, the use of hormone therapy may only be introducing potential cardiovascular toxicity which may be driving worse OS in this cohort. A recent systemic review addressed this topic and reported elevated cardiovascular disease including venous thromboembolism, myocardial infarction, stroke and other disorders in non-metastatic breast cancer patients treated with tamoxifen^40^.

Despite the reported benefit of RT for all patients, and specifically for those with luminal A/B (ER^+^) receptor-based surrogate subtypes and favorable disease, RT was significantly underused in this cohort. This seems to be a missed opportunity considering the available evidence supporting the use of definitive RT in such cases. To date most studies of definitive RT have involved elderly female patients. Interestingly, two such series with similar patient cohorts but different RT regimens reported drastically different OS rates. A study by Maher et al included 70 elderly female patients treated with definitive hypofractionated RT with tamoxifen and reported an excellent 3-year OS rate of 87%^5^. In contrast, Courdi et al evaluated the use of definitive SBRT with hormone therapy and found a 5-year OS rate of only 38%^4^. This difference may reflect the lower rate of treatment compliance and lack of regional nodal irradiation in the SBRT study. A more recent study comparing local control rates between breast-conserving therapy and definitive SBRT for elderly women with favorable disease features reported equivalent 7-year rates of cause-specific survival of 96.4%^3^. Thus although surgical resection is still preferred for BC treatment, some patients with favorable disease features, especially those who are >60 years old with ER^+^, cT1-2N0 disease, may be candidates for definitive RT with acceptable long-term survival outcomes.

Several inherent limitations of this NCDB study must be acknowledged. Aside from OS, the NCDB lacks other clinically relevant endpoints such as local-regional recurrence, disease-specific survival, patient-reported outcomes, and toxicity. Additional limitations include coding accuracy and the presence of confounding factors which may impact the reliability and generalizability of this analysis. Finally, even though the NCDB contains data on roughly 70% of cancer patients treated in the United States, the data are at the hospital level rather than the population level, and thus the findings are not necessarily generalizable to the entire US population^41^. Despite these limitations, this study represents the largest series reported to date of patients who did not receive surgery for BC and provides important insights into key socioeconomic factors that may affect access to surgery.

In conclusion, more than 4% of women with non-metastatic breast cancer in the United States do not receive surgery. Overall this study highlights the importance of several socioeconomic factors, including being Black, having low income, being uninsured or having public insurance, and having a low education level, that may act as barriers to optimal oncologic management with surgery. This information should function to bring such vulnerable patients to the attention of providers so that they can offer early intervention and additional social support to improve adherence to recommended treatments and enhance patients’ abilities to make informed decisions. This study further establishes a benchmark for survival expectations for patients who cannot or choose not to undergo surgery for breast cancer. We also suggest tailored treatment options for such patients that emphasize the use of chemotherapy for those with TNBC or HER2^+^ disease while favoring definitive RT for those with ER^+^ cancers. Overall our findings call for prospective evaluation of definitive RT for elderly patients with early-stage ER^+^ breast cancer who are not candidates for surgery.

## Methods

### Patient selection

This retrospective cohort study used data from the NCDB, a national hospital-based cancer registry co-sponsored by the American College of Surgeons and the American Cancer Society that includes clinical and socioeconomic data for roughly 70% of patients with newly diagnosed cancer^41,42^. We queried the NCDB for female patients aged 18 years or more with invasive primary BC diagnosed from 2004 to 2016 (and subsequently for those diagnosed in or after 2010, to account for HER2 status). Exclusion criteria were having stage IV, unknown, cT0, cTx or pTis -stage, unknown surgical status, recurrent disease or metastatic disease, and being male (Fig. 1).

### Statistical analysis

Descriptive statistics were used to summarize patient characteristics. Chi-square or Fisher exact tests were used to test for differences between categorical variables, and Wilcoxon rank-sum or Kruskal-Wallis tests were used to detect differences in continuous variables between groups^43^.

OS was measured from the time of diagnosis to the time of death or last follow-up among patients treated without surgery from 2010 through 2015 (*n* = 29,340) to capture receptor-based subtype surrogates. OS time for patients alive at the time of last contact was censored at the time. The OS distribution was estimated by the Kaplan–Meier method^44^, with log-rank tests used to test for differences in survival between groups^45^. Regression analyses of survival data based on the Cox proportional hazards model were conducted for OS outcome^46^. All tests were two-sided, and *P* values of <0.05 were considered statistically significant. All analyses were conducted using SAS (version 9.4, Cary, NC) and S-plus (version 8.04, TIBCO Software Inc., Palo Alto, CA) statistical software.

### Ethics

The University of Texas MD Anderson Cancer Center institutional review board deemed our analysis of this public database to be exempt from review because its constituent data have been anonymized and are compliant with the Health Insurance Portability and Accountability Act and thus was approved for completion.

### Reporting summary

Further information on research design is available in the Nature Research Reporting Summary linked to this article.

## Supplementary information

Supplementary Information

Reporting Summary

## Data Availability

The data generated and analyzed during this study are described in the following data record: 10.6084/m9.figshare.14618769^47^. The data underlying the study are clinical and socioeconomic data from the National Cancer Database (https://www.facs.org/quality-programs/cancer/ncdb). Interested parties can query the NCDB for female patients aged 18 years or more with invasive primary breast cancer diagnosed from 2004 to 2016. However, the data were used under license from the NCDB, and so applications to access the data must be made to the NCDB. The analyses of the data are contained in the SAS file ‘ncdbjune2019.sas7bdat’. These data are also not openly available as the Data Use Agreement between the authors and the NCDB states that NCDB approval must be sought before the authors can share the data. To request access to these data, contact the corresponding author.

## Code availability

The code used in this analysis is available upon request.
