# Supplementary material for: Evaluation of overall survival and barriers to surgery for patients with breast cancer treated without surgery: a National Cancer Database analysis
Source: NPJ Breast Cancer. 2021 Jul 5;7:87. doi: 10.1038/s41523-021-00294-w (PMC8257645; doi:10.1038/s41523-021-00294-w)
Supplement: Supplementary file 1 — Supplementary Information [file 41523_2021_294_MOESM1_ESM.pdf]

Supplementary Table 1. Patterns of Care for Patients Who Did Not Undergo Surgery

	Breast Cancer Subtype, No. (%)			
Treatment Modality	*All*<br>*N = 50,626 (%)*	*TNBC*<br>*N = 4,663 (%)*	*HER2⁺*<br>*N = 5,702 (%)*	*Luminal A/B*<br>*N = 21, 612 (%)*
**Chemotherapy**				
Yes	14,581 (28.8)	2,568 (55.1)	2,661 (46.7)	3,075 (14.2)
No	32,636 (64.5)	1,771 (38.0)	2,627 (46.1)	16,832 (77.9)
Unknown	3,409 (6.7)	324 (6.9)	414 (7.3)	1705 (7.9)
**Hormone Therapy**				
Yes	14,953 (29.5)	79 (1.7)	1,075 (18.9)	9,031 (41.8)
No	31,705 (62.6)	4,320 (92.6)	4,017 (70.4)	10,578 (48.9)
Unknown	3,968 (7.8)	264 (5.7)	610 (10.7)	2,003 (9.3)
**Radiation Therapy**				
Yes	3,689 (7.3)	453 (9.7)	366 (6.4)	848 (3.9)
No	45,704 (90.3)	4,087 (87.6)	5,160 (90.5)	20,137 (93.2)
Unknown	1,233 (2.4)	123 (2.6)	176 (3.1)	627 (2.9)

Abbreviation: TNBC, triple-negative breast cancer
Data for TNBC, HER2+ and Luminal A/B were based on the patients diagnosed in 2010-2016 because HER2 data became available in 2010 and onward

Supplementary Table 2. Multivariate Analysis of Factors Associated with Use of Radiation Therapy for Patients Who Did Not Undergo Surgery

Variable Reference	*Comparison*	*Odds Ratio*	*95% Confidence Interval*	*P Value*
**Age, years**				**<0.0001**
Per-year increase	Continuous	0.986	0.983–0.989	
**CDC Score**				**<0.0001**
0-1	2-3	0.73	0.61–0.88	
**Insurance Status**				
Private	Uninsured	0.73	0.60-0.89	**0.0015**
**Facility Location**				**0.041**
South	West	0.77	0.69–0.87	<0.0001
	Northeast	0.86	0.78–0.95	0.0032

Abbreviations: CDC, Charlson/Deyo comorbidity score; CC, Cancer Center; CP, Cancer Program
